# Knowledge, Attitudes, and Perceptions of Dental Assistants regarding Dental Asepsis and Sterilization in the Dental Workplace

**DOI:** 10.1155/2021/5574536

**Published:** 2021-06-16

**Authors:** Syed Sarosh Mahdi, Zohaib Ahmed, Raheel Allana, Francesco Amenta, Daniyal Agha, Mohammad Wasay Latif, Umer Daood, Carina Mehanna

**Affiliations:** ^1^Faculty of Dentistry, Jinnah Medical & Dental College, Sohail University, Karachi, Pakistan; ^2^Centre of Clinical Research, Telemedicine and Telepharmacy, School of Medicinal and Health Products Sciences, University of Camerino, Camerino, Italy; ^3^Athena Centre for Advanced Research in Health Care, Camerino, Italy; ^4^Dental Public Health Graduate, College of Dental Medicine, Columbia University, New York City, NY, USA; ^5^Department of Pediatrics & Child Health, Aga Khan University, Karachi, Pakistan; ^6^Division of Clinical Dentistry, School of Dentistry, International Medical University Kuala Lumpur, 126 Jalan Jalil Perkasa 19, Bukit Jalil 57000, Wilayah Persekutuan Kuala Lumpur, Malaysia; ^7^Postgraduate Program Department of Esthetic and Restorative Dentistry, School of Dentistry, Saint Joseph University of Beirut, Beirut, Lebanon

## Abstract

**Materials and Methods:**

A cross-sectional study was carried out. A 27-item prepilot tested close-ended questionnaire was designed and administered online to collect data on knowledge of asepsis, sterilization, instrument handling, disinfection, hand-hygiene practices, dental practice, age, education, and experience level from March 2020 to June 2020.

**Results:**

Out of 70 dental assistants, the majority were aged between 21 and 29 years (44.30%), more than two-thirds (85.41%) of the dental assistants were working in a hospital, while 14.29% were working in private clinics, only 7.1% had a diploma in the dental assistant program, and 74% had more than 2 years of experience in practice. Dental assistants working in private practice (76.30) had a higher mean knowledge scores compared to those working in hospital (74.25), while those with less than 2 years of experience (75.61) had a higher scores compared to those with 2–5 years of experience (73.96).

**Conclusion:**

Better compliance with recommended infection control and waste management practices is needed for all dental assistants. Continuing education programs targeting such awareness are vital to improve the management of hazardous waste practices among dental assistants.

## 1. Introduction

Dental professionals are exposed to several occupational hazards due to the nature of their work and proximity to their patients. These risk factors include exposure to microbial and chemical hazards [1]. The risk of occupational exposure to infectious diseases in dental practice is increased with lack of knowledge regarding infection control protocols and poor infection control practices [2]. Dental team is at a very high risk of exposure to infections caused by blood-borne pathogens like hepatitis B virus (HBV), human immunodeficiency virus (HIV), and hepatitis C virus (HCV) [1]. Dental work force is also highly susceptible to other pathogens like streptococci and many other diseases of viral and bacterial origin that colonize the mouth and respiratory tract [3, 4]. There are many routes of infection transmission in the dental office, including blood, bodily fluids, droplets, needle-stick injury, contaminated water sources from the dental units, and aerosols as well as indirect transmission which occurs through contact with contaminated surfaces and instruments [5–7]. This has led to increased concerns regarding infection control and cross-infection practices in dental settings, which has been investigated in many countries including North America and Europe [8–12].

The correct application of infection control protocols and precautions can significantly reduce the risk of cross infection in dental settings [1]. Compliance with infection control guidelines is paramount in breaking the chain of infection of communicable diseases and safe delivery of dental care [13]. The Center for Disease Control and Prevention (CDC) published the guidelines for infection control in dental settings in 1993 and most of the international guidelines are based on these guidelines, but they have changed and evolved over time [14]. These preventive infection control protocols were laid down primarily as a response of the outbreak of the human immunodeficiency virus (HIV) epidemic of the 1980s, as these protocols have been upgraded and revised regularly following their adaption over the years [14]. Following its worldwide adoption, these were called the universal precautions and were implemented globally to prevent the transmission of blood-borne diseases like HBV, HBC, and HIV [15]. The Occupational Safety and Health Administration (OSHA), which is a regulatory body in the United States, also based its blood-borne pathogen infection control standards on the universal precautions following their acceptance [15]. These guidelines were further expanded with recommendation that all work surfaces, instruments, and equipments should be cleaned and disinfected after coming into contact with blood, saliva, and other infectious materials [16]. Work surfaces should be disinfected before arrival of new patient and after completion of the procedure on each patient [16]. Vaccinations of the dental workforce were made mandatory where available as some forms of exposures are inevitable, as it provides protection against vaccine-preventable diseases [17]. The reuse of syringes, saline solution, and vials between patients has resulted in multiple outbreaks of hepatitis B and hepatitis C infections and hence was completely prohibited [17]. All these guidelines have ultimately increased compliance on aseptic methods during administration of parenteral medications [18–20].

Many studies have shown poor compliance with infection control protocols despite the issuance of such comprehensive infection control guidelines [21]. Moreover, this issue is of more concern in developing countries where such guidelines and protocols have not been well documented and established [22]. Many hospitals lack infection control training programs, and some have reported lack of awareness among allied health personnel [22, 23]. This research focuses on the infection control knowledge of dental assistants, particularly chairside dental assistants working in private clinics and hospitals. Dental assistants play a key role in the prevention of cross-infection and the majority of dental assistants responsible for carrying out such tasks are not certified in developing countries. We conducted this study to assess the knowledge, attitude, and perceptions for dental asepsis and sterilization among dental assistants working in Karachi, Pakistan.

## 2. Methodology

The study was authorized by the ethical Review Board of Jinnah Medical and Dental College, Karachi, Pakistan, following approval from participating sites. To protect the privacy and confidentiality of participants, the survey was kept anonymous with no identifiable information. Participation in the study was voluntary with no compensation, and all participants received information regarding the aims and purpose of the study prior to their participation.

A nonprobability convenience sampling was utilized to take the sample from an accessible population. The questionnaire created for this study included questions about asepsis and sterilization procedures. We divided the questionnaire into subcategories according to the procedure involved. The first group of questions were regarding presterilization; group 2, verification of biological processes involved in sterilization cycles; group 3, autoclave use. Category 4 questions were regarding documentation of the sterilization process. Twenty-seven questions were created largely tailored to the standard precautions advocated by World Health Organization's (WHO's) health governing standards and recommended guidelines for dental practitioners in the local disinfection and decontamination unit (LDU) HSE, 2012 (revised edition 2014), which has been adapted by many developed countries [24].

The questionnaire was validated via a convenience sample from a small number of dental assistant trainees (*n* = 24). The validity of the content was evaluated through expert opinion and further analysis was carried out. The ethical committee also suggested a few minor changes in the questionnaire. The final questionnaire showed average internal consistency (Cronbach's alpha 0.68) with participants taking on average 9.3 (±2.4) minutes to complete.

The findings of the pilot survey questionnaire were not included in the key report. The principal investigator wrote to a number of dental associations, requesting their assistance in having dental assistants complete the research questionnaire. The questionnaire was administered in English language as it is considered the official language in Pakistan. The questionnaire was administered personally and through referrals, an online link was forwarded to various dental establishments in Karachi. A total of 70 responses were received by the research team during this period from a total of 105 dental assistants approached. The response rate was found to be 66.6%.

There were 3 questions related to demographic variables and the dependent variable of knowledge was measured as a continuous composite score using 24 close-ended questions on a Likert scale (1 = not at all/never, 2 = very little/rarely, 3 = /sometimes, 4 = to a great extent/always), making 24 minimum and 96 maximum score. There were four independent categorical variables: age at four levels, health system affiliation with 2 levels (private practice or hospital), having a diploma in the dental assistant program as dichotomous (yes or no), and years of experience in practice at three levels.

### 2.1. Statistical Analysis

Data was analyzed using IBM SPSS version 24.0 with 95% confidence interval and 5% margin of error. The observed sample size was found to be 70. All variables were coded and entered in SPSS (descriptive statistics comprising of frequency and percentages to evaluate the responses). A factorial analysis of variance (ANOVA) was used to identify significant differences in the knowledge scores of various demographic groups, with *p*-value of “< 0.05” considered significant.

## 3. Results

Majority of the participants were aged between 21 and 29 years (44.30%). More than two-thirds (85.41%) of the dental assistants were working in a hospital setting. Only 7.14% had a diploma in the dental assistant program, although 74% had more than 2 years of experience in practice (Table 1).

Among knowledge-based self-administered questions, 58% of the dental assistants understood the correct use of alcohol-based hand rubs; however, only a few (17.1%) indicated applying at correct times. Approximately 33% of dental assistants indicated that they were trained in safe practices for the handling of sharp objects while 24.3% made sure that all instruments are washed and disinfected as specified in the practice protocol. Overall, 91.4% maintained and updated their awareness of infection prevention and preventive strategies periodically. As far as its practical implications were concerned, only 5% of them properly cleaned and dried the reusable items regularly. While 92.9% of the dental assistants indicated that they ensured the appropriate personnel proper validation and annual performance requalification for each sterilizer; but only 11.4% always ensured that the critical instruments have been labeled with batch control identification information before sterilization (Table 2).

**Figure 1:**
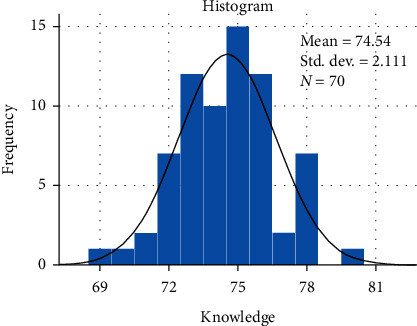
Histogram of knowledge score showing normal distribution.

**Figure 2:**
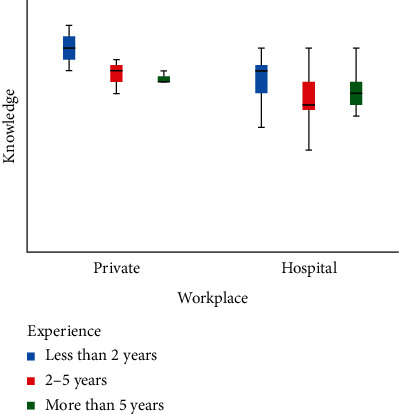
Box plot of knowledge score by health system affiliation and experience.

We performed factorial ANOVA and to determine if a parametric test ANOVA was appropriate to identify statistically significant knowledge score differences across independent groups (age, practice type, having a diploma, and years in practice), Shapiro-Wilk test of normality was used, which was insignificant (*p* > 0.05). It was also determined that the mean (74.54), median (75.00), and mode (75.00) were equal, further validating the assumption of using a parametric test. The histogram presented a normal distribution of knowledge score (Figure 1). Dental assistants working in private practice (76.30) had a higher knowledge score compared to those working in hospital. There was a significant difference in knowledge scores based on the level of experience in practice, those with less than 2 years of experience (75.61%) had a higher mean knowledge score compared to those with 2–5 years of experience (Table 3). The box plot showed that dental assistants working in private clinics with less than 2 years of experience had the highest knowledge score and the lowest variability. The dental assistants working in hospitals with 2–5 years of experience had the lowest knowledge score, but variability for this group was the highest (Figure 2).

## 4. Discussion

The results from this study showed that the knowledge of Pakistani dental assistants is limited in most areas of infection control, sterilization, waste disposal, and other aspects. Data regarding the actual number of practicing and registered dental assistants is difficult to find and hard to trust as most of the dental assistants working in dental clinics and hospitals are not certified. There is a dearth of education and training programs for dental assistants and nurses in Pakistan and this area is loosely regulated. Most people working as dental assistants and nurses in Pakistan get training during the job in either clinics or hospitals [25]. Dental assistants with less than 2 years of experience had a higher knowledge scores than those with higher experience. This finding may be consistent with the fact that infection control protocols and standards have been emphasized in recent years. We did not find any significant differences based on age and having a diploma in dental assistant program. One possible explanation for such finding could be the majority of dental assistants being younger than 40 years and very few (7%) had a dental diploma in a dental assistant program. With such limited variability across age, health system affiliation, and having a dental diploma, it is difficult to detect a difference in knowledge scores. A hospital-based cross-sectional study conducted in Japan found that compliance with infection control protocols had a significant correlation with age, dental department, patient frequency, knowledge, and openness to treat HIV/AIDS patients [26]. Such studies along with this study may provide a direction/basis for future research in this area.

Questions of our study focused on aspects of predisinfection, sterilization, waste management, compliance, and record keeping of infection control methods. We briefly discuss a logical path in four steps to highlight the process from where the dental instruments first arrive for disinfection, sterilization, and waste management. Studies of similar nature have demonstrated that immunization of dental teams toward bacterial and viral infections by disease-specific or nonspecific (e.g., gamma globulin) vaccines remains important [27]. One research in Melbourne, Australia, showed that just 5 out of 14 dental clinics processed and disposed of their hazardous waste according to the guidelines [28]. A research carried out in New Zealand observed that 24.45% of sharps discarded from dental clinics were discarded as household waste [29]. Waste contaminated with infectious materials should always be segregated in yellow exposure proof bags. The bags should be incinerated or go through the process of autoclave [30]. Only after carrying these procedures, infectious waste can be safely dumped into landfills [31]. Similarly, sharps can be stored in leak-proof bags and can either be incinerated or autoclaved before being placed into landfills [30]. Pharmaceutical wastage can be processed in brown plastic bags or bins and eliminated by encapsulation [30].

### 4.1. Predisinfection

The first step in the decontamination phase is cleaning that includes elimination of particles, debris, and microorganisms. Failure to extract noticeable debris or organic matter can impede the process of sterilization and disinfection [32]. Our research showed that that knowledge and application on predisinfection among dental assistants remained unsatisfactory and only 7.1% of the survey participants responded that they always cleaned and dried contaminated instruments before reuse. The instrument processing area should be divided into four distinct areas: cleaning and decontamination, packaging, sterilization, and storage [26]. In our study, only 11.4% participants admitted that all critical items are labeled and packaged with batch control identification before sterilization and only 5% of them properly cleaned and dried the reusable items regularly.

### 4.2. Sterilization

The primary purpose of sterilization is complete annihilation of all active microorganisms including bacterial spores. The most widely used method of sterilization is steam under pressure, which applies a high temperature of 121°C for 15–30 minutes or another similar but less commonly used method at 134°C for 3-4 minutes in an autoclave for removal of all viable microorganisms [33].

There has been a lot of research regarding infection control protocols recently, but evidence regarding the implementation of sterilization protocols remains very limited [34]. Our study indicates almost 90% of dental assistants updated their infection control, prevention, and sterilization knowledge every year.

### 4.3. Waste Management

Safe handling of hazardous waste is integral to adequate infection control [35]. Allied dental workers should be sufficiently trained in handling, storage, and disposal of hazardous waste. Waste laden with human tissue, blood, or bodily fluids such as swabs or dressings or other contaminated material must be clearly labeled as “clinical waste” and segregated from nonclinical waste [35]. Only half of the dental assistants in this study indicated that they fully ensure the safe handling of hazardous waste. According to Occupational Safety and Health Administration (OSHA), all dental personnel, including dentists who are at risk for occupational exposure, should receive initial and annual refresher training [36].

Used sharps include disposable syringes, needles, and anesthesia cartridges which are considered highly hazardous dental waste and are known to be associated with needle-stick injuries and disease transmission, particularly for dental assistants, who are responsible for the collection and disposal of such waste [36]. Such waste should be separated at the point of origin and deposited in a rigid, leak-proof, puncture resistant container labeled as “sharp's box” [29]. In the current study, only 32.9% respondents reported being trained in the handling and disposal of sharp objects. Other studies conducted in the Middle East region have found that 72% of primary health care government settings and 56% of private clinics had sharp containers for disposal of needles or other sharp instruments [37]. Findings from our study are relatively very poor in this domain. A color-coded yellow bag should ideally be used for collection of all infectious waste and the appropriate sections must be completed by the waste producer and the waste carrier [38]. Waste generated from amalgam should be collected and sealed separately until sent for recycling, as it contains mercury which could pose public health and environmental safety concerns [39].

Compliance with infection control protocols through validation, compatibility, and maintenance of dental instruments: a thorough analysis of the research on dental workers conformity with the Infection Prevention Guidelines [34] found that the findings of most of the research were focused on questionnaire data and the nature of the analysis were not accurate enough to produce meaningful results. In a certain limited number of direct observation trials, data were collected utilizing data collection methods that were not unique to dental practice [40, 41].

This contained descriptions of the physical setting, facilities, administrative procedures, expertise, and preparations undergone by the dental staff. One study indicated that data collection may be accredited as a procedure appraisal method (AD3) for use in general dental practice. But the usage of data collection methods, accompanied by optical inspection, has proven to be a significant barrier to the prompt and reliable recording of data into an online archive. The system was unable to interpret the handwritten text quite well. It enhanced the time needed for input results, since the text always had to be entered manually [40]. This leaves much room for improvement and more research needs to be carried out to the device methodology which records infection control and decontamination protocols. The concept of instrument procurement as an element of the decontamination and infection control process must be stressed to dental staff. Some of the devices are imported by phone from a dental retailer, with no respect to the accuracy of the infection prevention and decontamination instructions as given by the vendor. This method could be significantly enhanced if dentists were approved for particular products, which could then be seen as a recommendation while ordering instruments. Landmark British studies from Scotland showed that most dental devices critical to the disinfection and infection prevention cycle, such as ultrasonic baths and benchtop steam sterilizers, are not contracted for deployment or checked at sufficient intervals, as suggested in a variety of scientific publications [42]. The severe complexity of the sterilization procedure involves frequent monitoring of the device, checked at periodic intervals by adequate checking. The study observed that several practices which require subscription to third party testing and maintenance had poor paperwork to support the efficacy of these maintenance visits [42]. Relevant testing and maintenance of dental instruments remain a little discussed topic in the literature and there is a clear lack of guidance and awareness on this issue. Dental team needs to be given quality guidelines on how to improve this aspect of their practice to ensure good value for money and health protection.

## 5. Limitations

Our study had potential limitations, the generalizability of this study is questionable because of the convenience sample and the results cannot be generalized to all dental assistants working in Pakistan. Another major limitation of the study is the unequal sample sizes across various independent groups, particularly those working in private clinics and hospitals. Other limitations include control of confounders, and there are many other factors that affect perception, attitude, and knowledge of asepsis and sterilization, which are educational content, standardization of dental facilities, culture of an organization, stress, anxiety, and economic status. None of these factors were accounted for in the current research. The survey measures self-reported knowledge scores based on perceptions and attitudes of dental assistants, which cannot predict actual behavior. The knowledge score reported is subjective rather than objective.

## 6. Conclusion

Our study identified important data regarding asepsis and sterilization practices among dental assistants working in different healthcare settings in Karachi, Pakistan. This study may be considered as the first step in the process of needs assessment to highlight such a gap. Better compliance with recommended infection control and waste management practices is needed for all dental assistants. Continuing education programs promoting infection control awareness are vital to improve sterilization, cross-infection, and management of hazardous waste practices among dental assistants. Infection control and waste management training will not only improve the safety of all allied dental personnel but also help us maintain/restore a healthier environment.

## Figures and Tables

**Table 1:** Demographic characteristics of dental assistants (*n* = 70).

Characteristics	Count (%)
*Age*	
	
19–20 years	22 (31.40)
	
21–29 years	31 (44.30)
	
30–39 years	15 (21.40)
	
40–49 years	2 (2.90)
	
*Health system affiliation*	
	
Private practice	10 (14.29)
	
Hospital	60 (85.71)
	
*Had a diploma in dental assistant program*	
	
Yes	5 (7.14)
	
No	65 (92.86)
	
*Years of experience in practice*	
	
Less than 2 years	18 (25.70)
	
2–5 years	28 (40.00)
	
More than 5 years	24 (34.30)

**Table 2:** Knowledge, attitudes, and perception of dental asepsis and sterilization among dental assistants.

Select knowledge questions	Count (%)
*Correct techniques for use of alcohol-based hand rub*	
To great extent	41 (58.58%)
Somewhat	29 (41.42%)

*Application of hand rub techniques at the correct times*	
Always	19 (27.14%)
Sometimes	51 (72.86%)

*Do you ensure all critical items are packaged and labeled with batch control identification information before sterilization?*	
Always	08 (11.42%)
Sometimes	59 (84.28%)
Rarely	03 (4.30%)

*Do you ensure all instruments are cleaned and disinfected, as defined within the practice standards?*	
Always	17 (24.28%)
Sometimes	53 (75.72%)

*Do you maintain and refresh your knowledge on infection, prevention, and control measures annually?*	
Yes	64 (91.42%)
No	06 (8.58%)

*Ensure all contaminated reusable items properly cleaned and dried*	
Always	05 (7.14%)
Sometimes	65 (92.86%)

*Trained in safe practices for the handling and disposal of sharp objects*	
To great extent	23 (32.86%)
Somewhat	47 (67.14%)

*Ensure appropriate storage and handling of critical items to maintain their sterility until point of use*	
Always	05 (7.14%)
Sometimes	43 (61.42%)
Rarely	22 (31.44%)

*Ensure equipment and materials, which have been in contact with the patient's mouth, are handled appropriately*	
Somewhat	62 (88.58%)
Very little	08 (11.42%)

*Do you ensure validation and annual performance requalification are properly performed for each sterilizer?*	
Yes	65 (92.86%)
No	05 (7.14%)

**Table 3:** Analysis of variance for age, health system affiliation, diploma, and years of experience according to mean knowledge score.

Independent variables	Knowledge score (mean)	*p* value (<0.05)
*Age*		
		
19-20	75.23	
		
21–29	74.10	
		
30–39	74.33	
		
40–49	75.50	0.237
		
*Health system affiliation*		
		
Private practice	76.30	
		
Hospital	74.25	0.005
		
*Having diploma in dental assistant program*		
		
Yes	74.20	
		
No	74.57	0.709
		
*Years of experience in practice*		
		
Less than 2 years	75.61	
		
2–5 years	73.96	
		
More than 5 years	74.42	0.031

## Data Availability

The datasets generated and/or analyzed during the current study are available from the corresponding author on reasonable request.

## References

[1] Ayatollahi J., Ayatollahi F., Ardekani A. M (2012). Occupational hazards to dental staff. *Dental Research Journal*.

[2] Khader Y. S., Airan D. M., Al-Faouri I. (2009). Work stress inventory for dental assistants: development and psychometric evaluation. *Journal of Public Health Dentistry*.

[3] Milward M. R., Cooper P. R. (2007). Competency assessment for infection control in the undergraduate dental curriculum. *European Journal of Dental Education*.

[4] Kumar S., Sharma J., Duraiswamy P., Kulkarni S. (2009). Infection control practices among undergraduate students from a private dental school in India. *Revista Odonto Ciência*.

[5] Shah R., Collins J. M., Hodge T. M., Laing E. R. (2009). A national study of cross infection control: “are we clean enough?. *British Dental Journal*.

[6] Merchant V. A. (1991). Herpesviruses and other microorganisms of concern in dentistry. *Dental Clinics of North America*.

[7] Lin S. M., Svoboda K. K., Giletto A., Seibert J., Puttaiah R (2011). Effects of hydrogen peroxide on dental unit biofilms and treatment water contamination. *European Journal of Dentistry*.

[8] Bentley E. M., Sarll D. W. (1995). Improvements in cross-infection control in general dental practice. *British Dental Journal*.

[9] DiAngelis A. J., Martens L. V., Little J. W., Hastreiter R. J. (1989). Infection control practices of Minnesota dentists: changes during 1 year. *The Journal of the American Dental Association*.

[10] Gerbert B. (1987). AIDS and infection control in dental practice: dentists’ attitudes, knowledge, and behavior. *The Journal of the American Dental Association*.

[11] Gibson G. B., Mathias R. G., Epstein J. B. (1995). Compliance to recommended infection control procedures: changes over six years among British Columbia dentists. *Journal (Canadian Dental Association)*.

[12] Treasure P., Treasure E. T. (1994). Survey of infection control procedures in New Zealand dental practices. *International Dental Journal*.

[13] Singh A., Purohit B. M., Bhambal A., Saxena S., Singh A., Gupta A. (2011). Knowledge, attitudes, and practice regarding infection control measures among dental students in Central India. *Journal of Dental Education*.

[14] Computational Fluid Dynamics (2016). *Summary of Infection Prevention Practices in Dental Settings: Basic Expectations for Safe Care*.

[15] Computational Fluid Dynamics (1987). *Recommendations for Prevention of HIV Transmission in Health-Care Settings*.

[16] Computational Fluid Dynamics (1988). *Update: Universal Precautions for Prevention of Transmission of Human Immunodeficiency Virus, Hepatitis B Virus, and Other Bloodborne Pathogens in Health-Care Settings*.

[17] McCarthy G. M., Britton J. E. (2000). A survey of final-year dental, medical and nursing students: occupational injuries and infection control. *Journal (Canadian Dental Association)*.

[18] Department of Labor (1991). 29CFR Part 1910.1030, occupational exposure to bloodborne pathogens; final rule. *Federal Register*.

[19] Siegel J. D., Rhinehart E., Jackson M., Chiarello L. 2007 guideline for isolation precautions: preventing transmission of infectious agents in health care settings. *American Journal of Infection Control*.

[20] Thompson N. D., Perz J. F., Moorman A. C., Holmberg S. D. (2009). Nonhospital health care-associated hepatitis B and C virus transmission: United States, 1998–2008. *Annals of Internal Medicine*.

[21] Smith A., Dickson M., Aitken J., Bagg J. (2002). Contaminated dental instruments. *Journal of Hospital Infection*.

[22] Morris E., Hassan F. S., Al Nafisi A., Sugathan T. N. (1996). Infection control knowledge and practices in Kuwait: a survey on oral health care workers. *Saudi Dental Journal*.

[23] Sobayo E. I. (1991). Nursing aspects of infection control in developing countries. *Journal of Hospital Infection*.

[24] Bourgeois D., Dussart C., Saliasi I., Laforest L., Tramini P., Carrouel F. (2018). Observance of sterilization protocol guideline procedures of critical instruments for preventing iatrogenic transmission of creutzfeldt-jakob disease in dental practice in France, 2017. *International Journal of Environmental Research and Public Health*.

[25] Sheridan C., Gorman T., Claffey N. (2008). Dental nursing education and the introduction of technology-assisted learning. *European Journal of Dental Education*.

[26] Nagao Y., Matsuoka H., Kawaguchi T., Ide T., Sata M. (2008). HBV and HCV infection in Japanese dental care workers. *International Journal of Molecular Medicine*.

[27] Kohn W. G., Collins A. S., Cleveland J. L., Harte J. A., Eklund K. J., Malvitz D. M. (2003). Guidelines for infection control in dental health-care settings-2003.

[28] Cannata S., Bek M., Baker P., Fett M. (1997). Infection control and contaminated waste disposal practices in southern Sydney area health service dental clinics. *Australian Dental Journal*.

[29] Treasure E. T., Treasure P. (1997). An investigation of the disposal of hazardous wastes from New Zealand dental practices. *Community Dentistry and Oral Epidemiology*.

[30] Pankhurst C. L., Coulter W. A. (2017). *Basic Guide to Infection Prevention and Control in Dentistry*.

[31] De Jonge S., Boldingh Q., Solomkin J., Dellinger P., Egger M., Salanti G. Conference on prevention & infection control.

[32] Boyce R., Mull J. (2008). Complying with the occupational safety and health administration: guidelines for the dental office. *Dental Clinics of North America*.

[33] Sebastiani F. R., Dym H., Kirpalani T. (2017). Infection control in the dental office. *Dental Clinics of North America*.

[34] Gordon B. L., Burke F. J. T., Bagg J., Marlborough H. S., McHugh E. S. (2001). Systematic review of adherence to infection control guidelines in dentistry. *Journal of Dentistry*.

[35] Hashim R., Mahrouq R., Hadi N. (2011). Evaluation of dental waste management in the Emirate of Ajman, United Arab Emirates. *Journal of International Dental and Medical Research*.

[36] Chartier Y. (2014). *Safe Management of Wastes from Health-Care Activities*.

[37] Kurdy S., Fontaine R. (1997). Survey on infection control in MOH dental clinics, Riyadh. *Journal of Saudi Epidemiology Bulletin*.

[38] Directive CJOJoEU. 32/EU (2010) (2010). Implementing the framework agreement on prevention from sharps injuries in the hospital and healthcare sector, concluded by HOSPEEM and EPSU.

[39] Atesagaoglu A., Omurlu H., Ozcagli E., Sardas S., Ertas N. (2006). Mercury exposure in dental practice. *Operative Dentistry*.

[40] Smith A. J., Hurrell D., Bagg J., McHugh S., Mathewson H., Henry M. (2007). A method for surveying instrument decontamination procedures in general dental practice. *British Dental Journal*.

[41] Roebuck E. M., Strang R., Green I., Smith A., Walker J. (2008). The availability and content of dental instrument manufacturers’ decontamination information. *British Dental Journal*.

[42] Scotland N. (2004). Sterile services provision review group. *Survey of Decontamination in General Dental Practice*.

