# Supplementary material for: Knowledge, Attitudes, and Perceptions of Dental Assistants regarding Dental Asepsis and Sterilization in the Dental Workplace
Source: Int J Dent. 2021 Jun 16;2021:5574536. doi: 10.1155/2021/5574536 (PMC8225430; doi:10.1155/2021/5574536)
Supplement: Supplementary Materials — Questionnaire of the study is provided. [file 5574536.f1.docx]

**Knowledge, attitudes and perception of dental assistants regarding dental asepsis and sterilization in the dental workplace**

Where are you working right now?

1. In a private clinic
2. In a hospital

What age bracket do you fall under?

1. 18-20
2. 21-29
3. 30-39
4. 40-49
5. Do you have diploma in dental assistants program?
6. Yes
7. No
8. Do you understand and apply the correct techniques for use of alcohol based hand rub?
9. To a Great Extent
10. Somewhat
11. Very Little
12. Not at All
13. Do you apply these techniques at the correct times?
14. Always
15. Sometimes
16. Rarely
17. Never
18. Do you routinely practice other hand hygiene protective measures? (Such as disinfection by alcohol based hand sanitizers)
19. Always
20. Sometimes
21. Rarely
22. Never
23. Do you use personal protective equipment properly during all procedures?
24. Always
25. Sometimes
26. Rarely
27. Never
28. Are you trained in safe practices for the handling and disposal of sharp objects?
29. To a Great Extent
30. Somewhat
31. Very Little
32. Not at All
33. Do you ensure the safe handling of hazardous waste?
34. Always
35. Sometimes
36. Rarely
37. Never
38. Are you trained in minimizing the degree and extent of contamination within a contaminated zone?
39. To a Great Extent
40. Somewhat
41. Very Little
42. Not at All
43. Do you ensure all instruments are cleaned and disinfected, as defined within the practice standard?
44. Always
45. Sometimes
46. Rarely
47. Never
48. Do you ensure that the water in your practice environment, including your waterlines, is safe for dental patients?
49. Always
50. Sometimes
51. Rarely
52. Never

1. Are you aware of standard practice protocols regarding precautions for infection control, required in a dental clinic/hospital?
2. To a Great Extent
3. Somewhat
4. Very Little
5. Not at All
6. Do you ensure contaminated items for dispatch are decontaminated appropriately before dispatch?
7. Always
8. Sometimes
9. Rarely
10. Never
11. Do you ensure equipment and materials, which have been in contact with the patient’s mouth, are handled appropriately?
12. To a Great Extent
13. Somewhat
14. Very Little
15. Not at All
16. Have you ever reused disposable items (which are discarded after single use) on the patient?
17. Always
18. Sometimes
19. Rarely
20. Never
21. Do you ensure that reusable items are reprocessed properly, as appropriate for their intended use?
22. Always
23. Sometimes
24. Rarely
25. Never
26. Do you ensure an appropriate reprocessing area is designated for reprocessing procedures, to clean reprocessing flow?
27. To a Great Extent
28. Somewhat
29. Very Little
30. Not at All
31. Do you ensure all contaminated reusable items are properly cleaned and dried?
32. Always
33. Sometimes
34. Rarely
35. Never
36. Do you ensure all critical items are packaged and labeled with batch control identification information before sterilization?
37. Always
38. Sometimes
39. Rarely
40. Never
41. Do you ensure all reusable items are sterilized using a steam sterilizer with an appropriate cycle type?
42. Always
43. Sometimes
44. Rarely
45. Never
46. Are all packaged items processed in a steam sterilizer with drying capability?
47. Always
48. Sometimes
49. Rarely
50. Never
51. Do you ensure each sterilization cycle is appropriately monitored?
52. To a Great Extent
53. Somewhat
54. Very Little
55. Not at All
56. Do you ensure appropriate storage and handling of critical items to maintain their sterility until point of use?
57. Always
58. Sometimes
59. Rarely
60. Never
61. Do you ensure the appropriate performance tests for each piece of reprocessing equipment are carried out at the correct times?
62. Always
63. Sometimes
64. Rarely
65. Never
66. Do you ensure daily maintenance checks for reusable equipment?
67. To a Great Extent
68. Somewhat
69. Very Little
70. Not at All
71. Do you ensure validation and annual performance requalification are properly performed for each sterilizer by the appropriate personnel?
72. Yes
73. No
74. Are you aware of the procedures you must follow in the event of a sharp object injury?
75. To a Great Extent
76. Somewhat
77. Very Little
78. Not at All
79. Do you maintain and refresh your knowledge on infection, prevention and control measures annually?
80. Yes
81. No
